# Digital wear analysis and retention of poly-ether-ether-ketone retentive inserts versus conventional nylon inserts in locator retained mandibular overdentures: in-vitro study

**DOI:** 10.1007/s00784-024-05831-y

**Published:** 2024-08-06

**Authors:** Mahmoud Saleh Fayed, Nancy Nader Elsherbini, Bassem Mohsen, Reham Osman

**Affiliations:** 1 Department of Prosthodontics, Future University in Egypt, St. Teseen, New Cairo 1, Cairo Governorate, New Cairo, 11835 Egypt; 2 Department of Prosthodontics, Cairo University in Egypt, Giza, Egypt

**Keywords:** Overdenture, Digital, Wear analysis, Dental implants, Retention, PEEK, Nylon, Implant overdentures

## Abstract

**Objective:**

this study aimed to digitally compare wear behavior and retention between PEEK and nylon retentive inserts used in locator-retained, mandibular implant overdentures when attachment design and size were standardized.

**Materials and methods:**

A total of sixty-four inserts (32 PEEK and 32 nylon inserts); were picked-up in implant overdentures. Overdentures of both groups were submerged in artificial saliva and mounted to chewing simulator. After 480,000 chewing cycles (equivalent to 2 years of clinical use) all inserts were scanned by scanning electron microscope (SEM), then all acquired images were digitally analyzed by software to detect and compare quantitative and qualitative changes of inserts in both groups. On the other hand, retention of both groups was measured by universal testing machine and the collected data was statistically analyzed using one-way Analysis of Variance (ANOVA) test with significance level set at *P* ≤ 0.05.

**Results:**

PEEK inserts showed significantly higher mean retentive values compared to the nylon inserts in the control group. Also, PEEK retentive inserts exhibited statistically lower mean wear values than the control group *P* ≥ 0.000. Qualitative investigation revealed significant and more pronounced changes in the surface roughness of nylon inserts compared to PEEK ones.

**Conclusions:**

Regarding retention, wear behavior and dimension stability, PEEK can be recommended as retentive insert material in cases of locator-retained mandibular implant overdentures.

**Clinical relevance:**

PEEK inserts offer enhanced retention, reduced wear, and greater dimensional stability over two years time interval. Clinically, this reduces prosthodontic maintenance and adjustments, improving patient satisfaction and long-term prosthetic success.

## Introduction

Mandibular implant overdentures represent an established treatment modality for the management of edentulous predicament resolving retention and stability problems associated with conventional mandibular complete dentures [1, 2].

Various types of attachment systems are used to retain implant overdentures. Among these attachment systems, stud attachments have increased popularity over other types of attachments due to favorable stress distribution, simple fabrication procedures and the ease of aftercare [3].

Locator is a subgroup of stud attachments with a self-aligning design feature aimed at overcoming the prosthetic problems associated with malangulated implants and patients with reduced inter-arch spaces [4, 5]. Furthermore, dual retention feature of the locator attachments provided by the outer and inner insert surfaces provides greater retention surface area compared to other attachment types and ensures a long-lasting performance [4].

However, wear of the retentive inserts and reduced retention over time are inevitable findings and are reported to be the most common prosthetic complications associated with long-term use of implant overdentures. Therefore, regular maintenance is of utmost importance to ensure successful long-term outcome [6, 7]. The retentive insert design, chemical structure, as well as the physical and mechanical properties of the retentive insert are all influential factors on wear and retentive properties of inserts and subsequently on the frequency of maintenance events that will be needed [7].

Many materials have been proposed for construction of attachment inserts such as polyamides, polyvinylsiloxane, PEEK (polyether ether ketone), polyoxymethylene and silicones [8].

Polyamides (nylons) were mainly used in construction of retentive inserts for different dental attachment systems due to high mechanical strength, good fatigue resistance, ease of machinability and excellent wear resistance of the material [9].

Nevertheless, with the recent advancements in the field of biomaterials, new polymers with enhanced physical and mechanical properties were introduced to replace traditional materials. PEEK represent one of such polymers for the application in dental field generally and field of Prosthodontics specifically [10].

PEEK is a thermoplastic polymer with semi-crystalline structure. The crystallinity of material accounts for PEEK superior mechanical, physical and thermal properties, which accounts for the popular use of the material as a high performance matrix material [11–13].

However, there is scarcity of data on retention values, and its correlation to wear behavior and wear mechanisms of recently introduced PEEK retentive inserts. Therefore, the aim of this study was to digitally evaluate the wear behavior and the retention of PEEK retentive inserts in cases of locator-retained mandibular overdentures and compare it with the more commonly used nylon retentive inserts.

The null hypothesis was that there is no difference in the amount of retention and wear behavior between conventional nylon inserts and the recently introduced PEEK inserts in cases of locator-retained mandibular overdentures.

## Materials and methods

### Study settings

This in vitro study aimed to compare the retentive and wear behavior of two materials used for retentive inserts: Polyetheretherketone (PEEK) as the intervention group and nylon as the control group. Each group will consist of 8 retentive inserts, making the total sample size 16 inserts per time interval. The study was designed to simulate 2 years to clinical, with follow-ups at four time intervals: T1 = 6 months, T2 = 12 months, T3 = 18 months, and T4 = 24 months. At each interval, retention will be measured using a universal testing machine, and the wear of inserts will be assessed both quantitatively and qualitatively via scanning electron microscopy.

### Fabrication of models

Two identical epoxy models of a completely edentulous mandible; ACP class I, with 6 mm width and 12 mm height were fabricated using a silicon mold of a ready-made ideal stone model. One model was scanned twice; the first scan was done using an intra-oral digital scanner (Medit, i500) to generate the Standard Tessellation Language (STL) files, while the second scan was performed using CBCT machine (Sirona GALILEOS) to generate the DICOM file for the generation of virtual model and the design of surgical guide.

### Construction of surgical guide and insertion of implants

Both STL and DICOM files were imported and merged by digital software (BlueSky, 2019) to construct a 3D virtual model upon which a surgical guide was designed.

Implants position was planned in the interforaminal region between lateral incisors and canine bilaterally at a distance of 23 mm (11.5 mm from the midline at each side) where the parallelism between implants was ensured.

Implants diameter was chosen to be 3.8 mm according to the ridge width and the implant length was 10.5 mm. The diameter of surgical guide sleeve was 4 mm.

The guide was then printed in clear resin (EPAX 3D resin, EPAX, USA) using 3D printer (Epax X10, EPAX, USA). After finishing and polishing, the stability of guide was verified on the model by alternating finger pressure on both sides of guide and then it was fixed in position by means of reversible glue (Titebond, USA).

The implants (Biohorizon implant, USA) were then inserted in preplanned position and the model was inserted in warm water bath (≥ 45^0^ C) for 2–3 min to reverse the bonding glue and allow removal of the guide.

Locator abutments (Humanna, PEEK Loc, Germany) were loaded on both implants in each group and tightened by torque wrench at 15 Ncm as recommended by the manufacturer. Then, two overdentures (one for each study group) were constructed according to the standard protocol [14].

PEEK inserts (Humanna, PEEK Loc, Germany) are available only in retention value of 5 Ibs, and thus pink nylon inserts with the same amount of retention (5 Ibs equivalent to 22.24 N) were selected from the wide array of nylon inserts available.

Abutment undercuts were blocked-out using provided rubber spacer and any further undercuts were blocked out using teflon. Retentive inserts were then inserted on locator attachments and picked-up on the fitting surface of overdentures. The attachment retentive inserts were picked-up using auto polymerized acrylic resin following standardized protocol. Excess acrylic was then removed and the surface was finished and polished [15].

After that the geometric center of the dentures was determined as described in previous literature to standardize a point from which dentures can be pulled during the cycles of removal [16].

### Aging of retentive inserts

The aging of retentive inserts was simulated by using both a chewing simulator to replicate chewing cycles and a universal testing machine to simulate insertion and removal cycles. Both types of cycles are crucial for a comprehensive clinical simulation of insert usage.

**Fig. 1:**
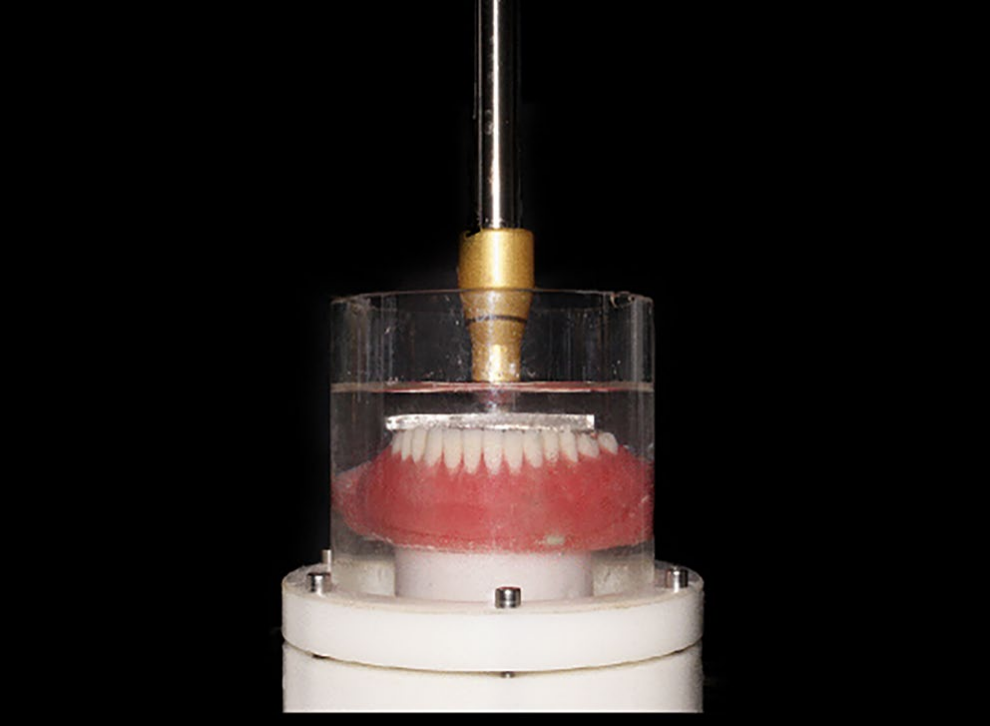
Model connected to a chewing simulator

For the chewing cycles, each model was connected to a chewing simulator (CS-4.4; SD Mechatronic, Germany). The machine parameters included a 5 mm vertical path, 60 mm/sec speed, 0.5 mm horizontal path, 1.6 Hz frequency, and 68.6 N forces. Artificial saliva was used to replicate oral conditions [17]. The overdentures underwent cycles to simulate different durations of clinical use: T0 (0 cycles), T1 (120,000 cycles, equivalent to 6 months), T2 (240,000 cycles, equivalent to 12 months), T3 (360,000 cycles, equivalent to 18 months), and T4 (480,000 cycles, equivalent to 24 months). (Figure 1)

For the insertion and removal cycles, each model was tested using an Instron universal testing machine (Model 3345) with a speed of 50 mm/min and a 2.5 mm dislodgement range (0.17 Hz), while immersed in artificial saliva [18]. The overdentures underwent cycles to simulate clinical use durations: T0 (0 cycles), T1 (720 cycles, equivalent to 6 months), T2 (1,440 cycles, equivalent to 12 months), T3 (2,160 cycles, equivalent to 18 months), and T4 (2,880 cycles, equivalent to 24 months).

### Measurement of retention

Universal testing machine (Instron, England, Model 3345) with a 500 N load cell at a crosshead speed of 50 mm/min, was used to measure retention values at different time intervals [15].

Following the completion of chewing cycles at different time intervals, while the cast models and the overdenture were still submerged in artificial saliva, ten linear dislodgement slides were performed perpendicular to the occlusal plane. The forces were recorded using compatible computer software (Bluehill Universal, Instron, England) and the resultant data were compared to the recorded data at (T0) to calculate the difference in the recorded retention values between different time intervals.

### Digital wear analysis

At T0, new unused inserts; one from each group was scanned from top and cross-sectional views by scanning electron microscope (SEM) (Quanta FEG 250 Scanning electron microscope, USA) to establish reference points to which results of subsequent scan data following different chewing cycles could be compared.

**Fig. 2:**
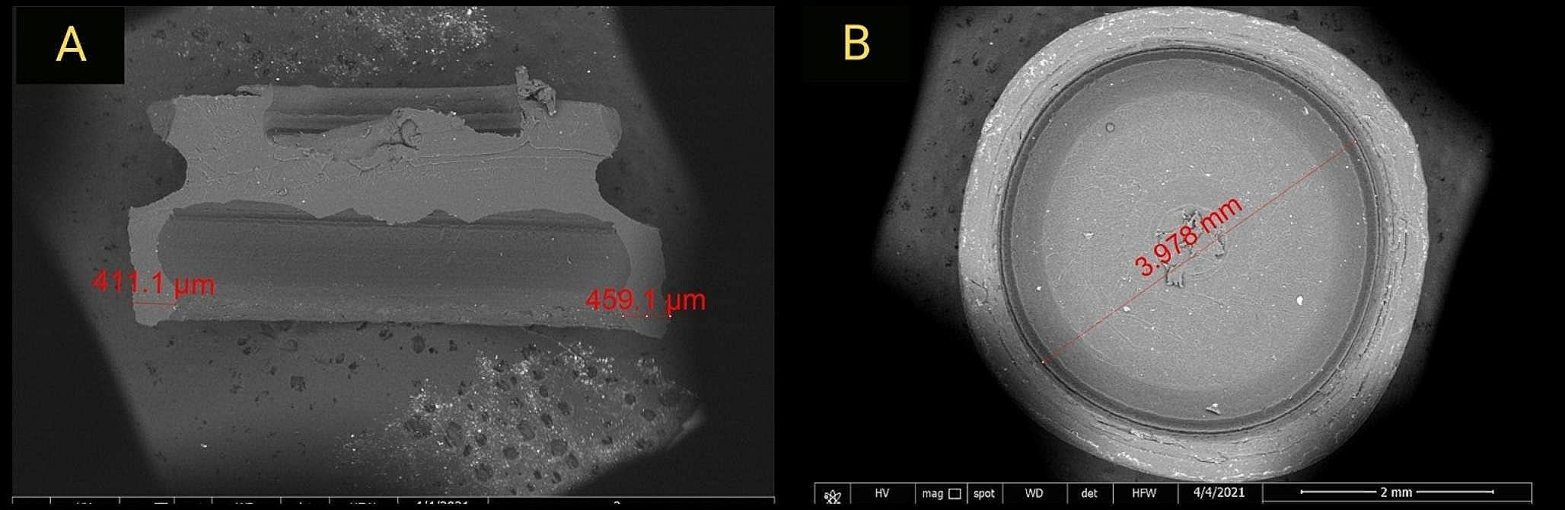
SEM image showing measurements of: **A**. Inserts’ wall thickness from cross-sectional view. **B**. Inserts’ inner diameter from top view

Scans of top view were used to measure inner diameter, while scans of cross section views were used to measure depth and thickness of insert walls engaging the abutment undercuts (Fig. 2).

Scanning parameters were standardized in all samples at different time intervals by fixation of magnification power at x55, working distance (WD) at 11.8 mm, high voltage (HV) at 20.00 kV and horizontal field width (HFW) at 7.74 mm.

At each of the following time intervals, new inserts were used and were subjected to chewing cycles. Following the completion of chewing cycles at each interval, inserts were rescanned form top and cross-sectional views, and was compared to T0 reference scans for wear analysis by detecting the amount and pattern of dimensional changes in the retentive inserts. A total number of sixty-four inserts were used for both groups; thirty-two per group (8 inserts per each time interval for each study group).

**Fig. 3:**
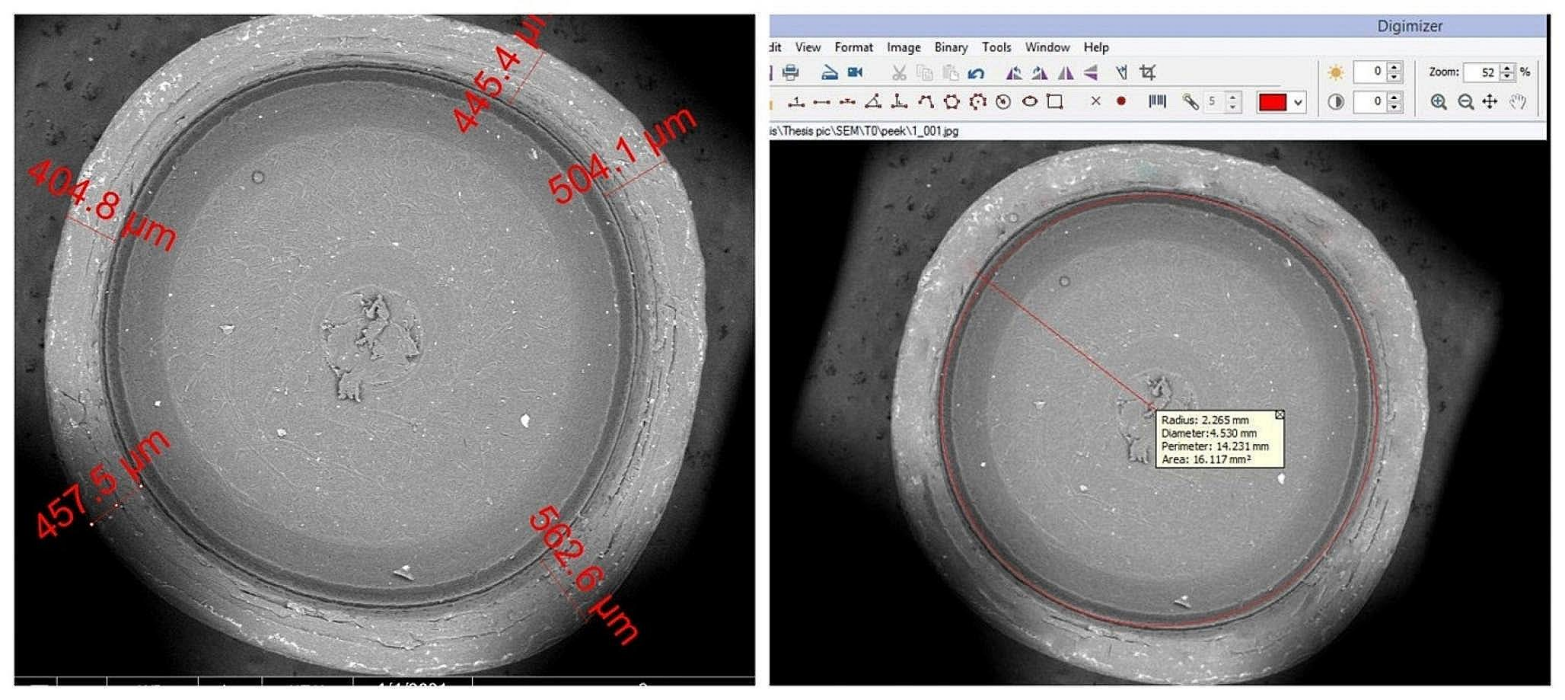
SEM image showing: **A**. Conversion of image pixels into measurable units (um) by Smartscan software. **B**. Measuring inner inserts diameter by Digimizer software

Wear analysis was performed both qualitatively and quantitatively. Quantitative assessment was done by measuring the amount of dimensional changes in diameter, wall thickness and depth of inserts. Quantitative wear analysis was performed by software (SmartSCAN, Epson, USA) to convert the pixels of SEM images into measurable units (millimeters) as seen in Fig. 3a. Digimizer, software was used to calculate the inner diameter based on Smartscan measuring scale as seen in Fig. 3b.

**Fig. 4:**
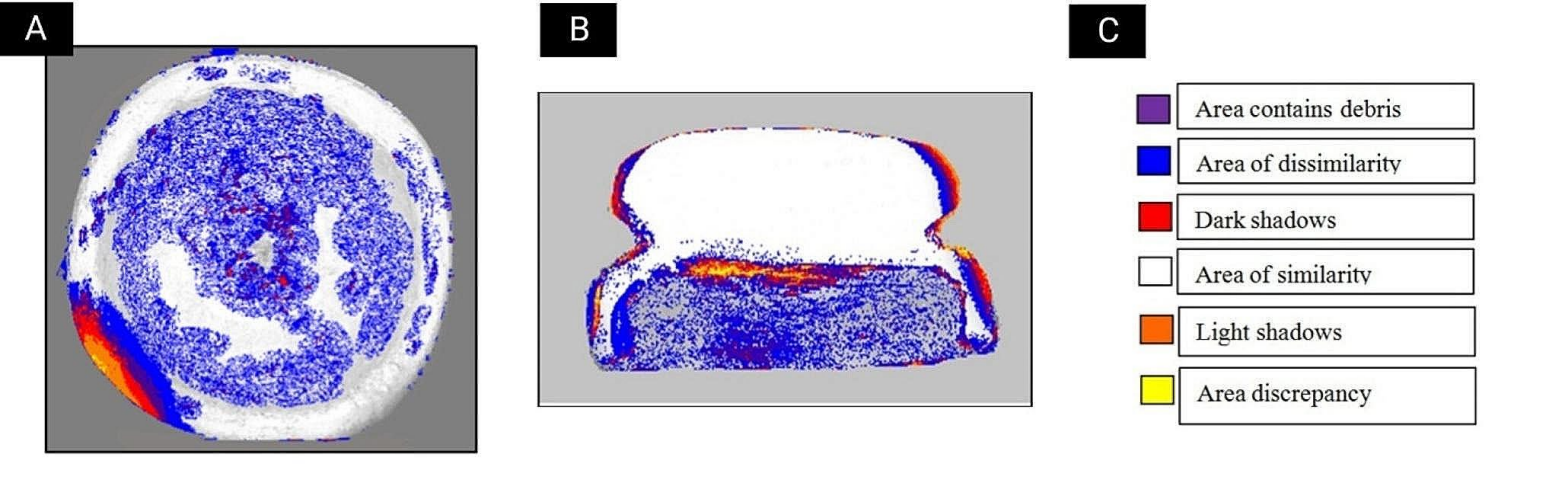
Qualitative wear analysis using thermal mode image highlighting changed areas in nylon inserts between T0 as reference images to other images from different time intervals. **A**. Top view comparison. **B**. Cross-section view comparison. **C**. Color scheme

Qualitative analysis was performed digitally by the evaluation of the changes of inserts surface morphology at different time intervals using specialized computer software (ImageDiff, version 1.2, USA). The photos were cropped to exclude any misleading background information that can result in the generation of false results. The test scans were then superimposed and compared with reference scan images at (T0) by pixels subtraction technique. ImageDiff software further allows the generation of color-guided maps of scanned inserts that enhances the qualitative analysis and interpretation of the results. (Fig. 4)

### Statistical analysis

Data were described using minimum, maximum, mean and standard deviation with 95% CI of the mean for each group. Data were explored for normality using Kolmogorov-Smirnov test and Shapiro-Wilk tests and both showed parametric (normal) distribution. Independent Sample t-test was used to compare parametric data of wear and retention between the different study groups. Comparisons were carried out between more than two independent normally distributed subgroups using one-way analysis of variance (ANOVA) test. Post-hoc multiple comparisons were done using Bonferroni method, clustered bar charts with 95% confidence interval and error bars were generated. An alpha level was set to 5% with a significance level of 95%, and a beta error accepted up to 20%. Power of study of 80% was considered during sample size calculation. Statistical analysis was performed with IBM SPSS Statistics Version 21 for Windows.

Based on previous studies that evaluated wear of overdenture resilient attachments, the minimum required sample size was found to be 8 inserts per group (number of groups = 2) to detect an effect size (two tails) of 1.508 in the wear of PEEK inserts compared to nylon inserts (primary outcome) [18–20].

The sample size was calculated using **G-Power** version 3.1.9.2 [21].

## Results

At T0, statistical analysis revealed no significant difference between the two groups in retention and wear values. Thus, at T0, all inserts were standardized and exhibited the same amount of retention and wear values in the inserts’ dimensions, and all changes recorded were related to the inserts’ material.

For the retention values, at (T1 = 120,000 chewing cycles), PEEK group showed significantly higher values compared to nylon inserts. This difference increased over time and was peaked at (T2 = 240,000 chewing cycles) where PEEK inserts retentive values were nearly twice as that of nylon. While at (T3 = 360,000 chewing cycles), there was a significant sudden increase in retention of the nylon group compared to the PEEK group (Table 1). At T4 = 480,000 chewing cycles, there was a re-drop in retention values of nylon inserts and an increase in the PEEK group. The difference was statistically significant as seen in Table 1.

**Table 1:** Descriptive statistics of the mean, min and max retention values for both groups at different time intervals and results of independent samples (Student’s) t test for the comparison of average insert retention between the two groups

Time of measurements	Mean ± standard deviation		*P*-value
	Nylon (Newton)	PEEK (Newton)	
T0	59.64 ± 1.22	60.80 ± 1.65	0.089
T1	45.28 ± 1.27	50.60 ± 2.44	0.000*
T2	24.23 ± 1.01	41.51 ± 1.86	0.000*
T3	37.02 ± 2.01	27.68 ± 1.23	0.000*
T4	14.12 ± 0.83	20.71 ± 1.31	0.000*

*: Significant

For wear assessment, at T0, no significant difference was found in dimensions and surface morphology of inserts between the two groups so that any subsequent recorded changes would be related to inserts’ wear behavior.

Quantitative wear analysis showed no significant difference in the inserts’ dimension between the two groups at T1 and T2. At T3 and T4 the diameter of PEEK inserts was significantly smaller and depth was significantly shallower compared to that in the nylon group (Tables 2 and 3).

**Table 2:** Descriptive statistics of the mean, min and max values of inserts’ diameter for both groups at different time intervals and the results of independent samples (Student’s) t test for the comparison of average diameter (mm) between the two groups

	Average Diameter		Test of significance *P*-value
	PEEK inserts *n* = 8	Nylon Inserts *n* = 8	
(T0) (mm) Mean ± Std.	4.076 ± 0.066	4.227 ± 0.190	*p* = 0.053
(T1) (mm) Mean ± Std.	4.076 ± 0.066	4.227 ± 0.190	*p* = 0.053
(T2) (mm) Mean ± Std.	4.103 ± 0.086	4.195 ± 0.095	*p* = 0.061
(T3) (mm) Mean ± Std.	4.051 ± 0.112	4.645 ± 0.082	*p* = 0.000*
(T4) (mm) Mean ± Std.	4.119 ± 0.105	4.409 ± 0.092	*p* = 0.000*

*: Significant

**Table 3:** Descriptive statistics of the mean, min and max values of inserts’ depth for both groups at different time intervals and the results of independent samples (Student’s) t test for the comparison of average depth (mm) between the two groups

	Average Insert Depth		Test of significance *P*- value
	PEEK inserts *n* = 8	Nylon Inserts *n* = 8	
(T0) (mm) Mean ± Std.	0.959 ± 0.030	0.948 ± 0.012	*P* = 0.320
(T1) (mm) Mean ± Std.	0.963 ± 0.019	0.955 ± 0.013	*P* = 0.373
(T2) (mm) Mean ± Std.	0.963 ± 0.019	0.963 ± 0.014	*P* = 0.343
(T3) (mm) Mean ± Std.	0.903 ± 0.036	1.101 ± 0.051	*P* = 0.000*
(T4) (mm) Mean ± Std.	0.925 ± 0.040	1.107 ± 0.013	*P* = 0.000*

*: Significant

**Fig. 5:**
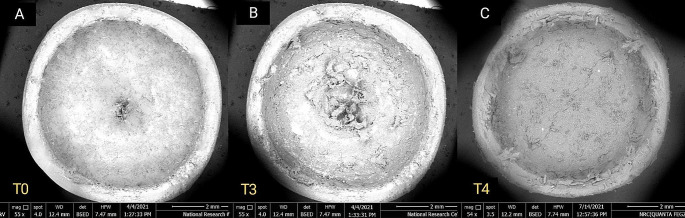
Top-view SEM images showing significant qualitative changes in inserts’ surface texture at different time intervals. **A**. Nylon insert at T0 **B**. Nylon insert at T3 **C**. Nylon insert at T4

**Fig. 6:**
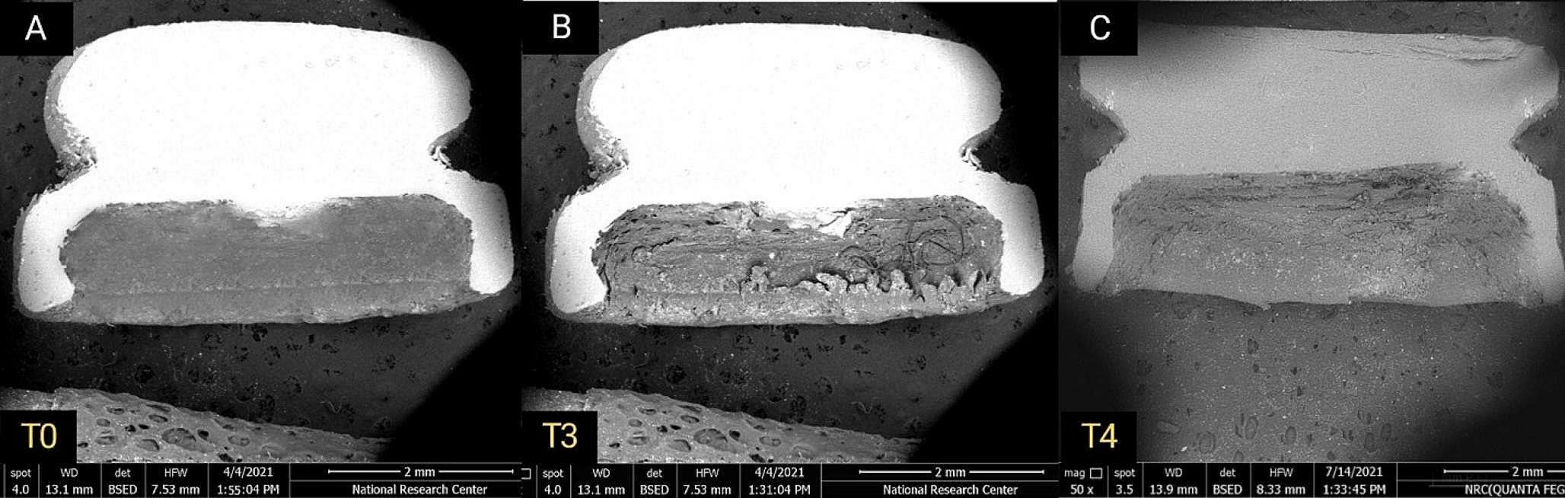
Cross section-view SEM images showing significant qualitative changes in inserts surface texture at different time intervals. **A**. Nylon insert at T0 **B**. Nylon insert at T3 **C**. Nylon insert at T4

**Fig. 7:**
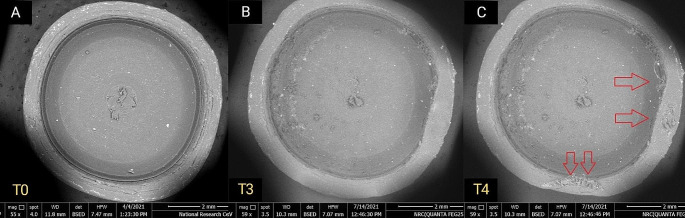
Top view SEM images showing minor qualitative changes in inserts surface texture at different time intervals as marked with red arrows. (**A**) PEEK insert at T0 (**B**) PEEK insert at T3 (**C**) PEEK insert at T4

For qualitative wear analysis, PEEK inserts showed minor morphological changes in inserts’ outline and thickness compared to nylon inserts at the same time interval as seen in Figs. 5, 6 and 7.

Wear pattern of nylon inserts was continuous, progressive and more extended while that of PEEK inserts was less progressive and less extended.

## Discussion

The aim of the current study was to evaluate the amount of retention and wear behavior of PEEK inserts and compare it to conventionally used nylon ones in cases of locator-retained mandibular implant overdentures when attachment design and size were standardized. Clinically, wear and the need for the inserts replacement and/or observable loss of retention are reported to occur in the majority of overdenture attachment systems within the initial 1-1.5 years of service [15, 22–25].

The null hypothesis was rejected, the results of the current study showed a difference in both retention and wear behavior between conventional nylon and the recently introduced PEEK inserts in cases of locator-retained mandibular overdentures at different evaluated time intervals.

The findings of this study revealed that PEEK inserts had significantly higher retention values at T1 and T2 compared to the inserts of the control group despite the fact that the quantitative wear analysis revealed no significant difference in wear values between the inserts of the two groups. Nevertheless, qualitative analysis revealed significant dimensional changes in the nylon group presented by significant decrease in wall thickness, loss of uniformity of original inserts’ shape (Figs. 5 and 6) Accumulation of wear debris resulted in surface roughness of inner inserts surfaces, and asymmetry of inserts walls. These changes were more prominent in the nylon group compared to PEEK inserts and were more pronounced in T2 compared to T1 in both groups (Fig. 6; Table 4) Continuous decrease in nylon inserts’ wall thickness resulted in loss of continuity of inserts and possible decrease in retention properties which can be clinically reflected in the form of increased post-insertion maintenance requirements [26–28]. This reflects the importance of combining both quantitative and qualitative analysis when evaluating the wear behavior of any material. In partial agreement with our findings, Wichmann et al., and Tehini et al. found no significant difference in inserts’ dimensions (diameter and depth) upon quantitative evaluation of wear behavior of both PEEK and nylon inserts at T1 and T2 time intervals (Tables 2 and 3) Contradictory to the later study, significant difference in inserts’ dimensions between PEEK and nylon inserts at **T3** and **T4** time intervals were observed in current study (Tables 2 and 3) and could be attributed to the difference in number of aging cycles which was employed in both studies and/or due to the difference in the material composition of the inserts used in their study and consequently the observed wear behavior [29, 30].

**Table 4:** Descriptive statistics of the mean, min and max values of inserts’ wall thickness for both groups at different time intervals and the results of independent samples (Student’s) t test for the comparison of average wall thickness (mm) between the two groups

	Average Wall Thickness		Test of significance *P*- value
	PEEK inserts *n* = 8	Nylon Inserts *n* = 8	
(T1) (mm) Mean ± Std.	0.462 ± 0.020	0.503 ± 0.032	*P* = 0.007*
(T2) (mm) Mean ± Std.	0.454 ± 0.020	0.496 ± 0.034	*P* = 0.009*
(T3) (mm) Mean ± Std.	0.430 ± 0.015	0.448 ± 0.015	*P* = 0.032*
(T4) (mm) Mean ± Std.	0.418 ± 0.014	0.400 ± 0.031	*P* = 0.156

*: Significant

At T3 time interval, equivalent to 1.5 year of clinical use, nylon group showed a sharp increase in retention which was also significantly higher than that of PEEK inserts (Table 1) This finding can be attributed to wear pattern of nylon inserts observed at such time interval. Similarly, quantitative wear analysis showed an increase in the depth and a decrease in the diameter (Tables 2 and 3) of the inserts thus resulting in increased abutment friction and thereby improved retention values recorded in nylon group [24, 25]. Contradictory to our findings, in an in vitro study Abdelaziz et al. reported significantly reduced retention of PEEK compared to nylon inserts of locator attachments at T3 time interval. These contradicting results may be due to the fact that the attachment system (Zest anchor F-Tx) that was used in the later study is originally designed for the use with fixed prostheses rather than with removable overdentures. Frequent insertion and removal of such an attachment not intended for daily removal by the patient may have resulted in the observed loss of retention of PEEK inserts at T3 time interval highlighting the importance of proper attachment selection as per manufacturer’s instructions [15].

At T4 time interval equivalent to 2 years of clinical use, PEEK inserts maintained significantly higher retention values compared to the nylon ones, which showed re-drop in retention values (Table 1). This finding was consistent with both the quantitative and qualitative results of wear analysis which revealed continuous, progressive and more extensive wear of nylon inserts at T4 compared to PEEK ones (Fig. 7). Both cross-section and top view scans showed profound changes in depth and wall thickness at the periphery of the nylon inserts. This observed wear pattern was different from what was described by Tehini et al., and Rabbani et al., [31, 32] where they found increased wear expression on the central stud rather than on the outer ring of the plastic insert. The difference in the observed wear pattern could be attributed to the fact that in the later described studies, qualitative wear analysis was mainly dependent on analysis of top view scans. This further stresses the importance of combining both top and cross-section scan views during the qualitative process of wear analysis as was the case in the current study.

To the authors’ best knowledge, the study at hand is the first study in literature to digitally evaluate wear behavior of prosthetic inserts both qualitatively and quantitatively and to correlate these findings to the retentive behavior of the inserts and thus enabling the prediction of the load of prosthetic maintenance in an actual clinical setting. Qualitative analysis can better demonstrate the relationship between dimensional changes and surface wear patterns of these attachments; an information which is almost lacking in the literature [18]. Quantitative measurements provided data regarding the amount of wear at different time intervals. Furthermore, a novel method was also used for the wear analysis implementing the use of both scanning electron microscope images and different automated image matching softwares. Wear analysis was further performed on both top view and cross-sectional scans of the inserts. Top view scans allowed the measurement of dimensional changes in the inner diameter and detection of any changes in shape or form of the inserts due to wear process [32, 33]. The cross-section scans enabled the accurate measurements of the depth and the thickness of insert walls engaging the abutment undercuts. ^19,31^

All the softwares employed in this study ensured accurate and reliable quantitative and qualitative analysis of wear process and provides a highly standardized method of measurements [32]. **SmartScan software** can standardize image scale for captured SEM photos. This was considered essential specifically during the qualitative analysis to facilitate examination of photos by different softwares as any difference in images scale would have resulted in misleading data [32]. ImagDiff software was used for qualitative wear assessment as it compares scanned photos at different time intervals to a reference photo based on the standardized image scale generated by SmartScan software. Detection of surface changes was done automatically by the ImagDiff software through images superimposition and pixel subtraction. This was important to overcome human errors and viewer bias. ImagDiff software is also capable of generating a color guide to facilitate the interpretation of results and enable the qualitative analysis. Quantitative wear measurement was performed using **Digimizer** software which is reported to be a valid and a reliable measurement method [34].

The in-vitro nature of this study is acknowledged; however, it was preferred over in vivo study design for the purpose of this study. Changing inserts every 6 month for two groups of patients would have been a time-consuming process that requires high patients’ commitment and cooperation. In vitro study design enabled the standardization of all study variables except for the factors under investigation. Another declared limitation of the study would be the linear direction of insertion and removal forces that was applied. Rather than the combined vertical and horizontal force directions typically encountered in clinical settings. Further, the mucosa was not simulated. Soft tissue mimics cannot replicate the actual clinic situation; it does not deteriorate nor does it regenerate to adapt to the denture base as the in vivo situation. After several chewing cycles, the simulated mucosa can wear down and undergo uneven dimensional changes. This deterioration could potentially affect the peripheral seal and introduce confounding variables into the results. It was thus the authors’ choice not to simulate the oral mucosa. The universal testing machine that was used to perform insertion and removal cycles allowed for automation and standardization of removal process only [31, 35].

The geometric center of the models was determined to standardize a point from which the dentures can be pulled during the removal cycles. This enabled the dentures of both groups to be pulled in the same the direction and with the same amount of force [16]. On the contrary, the removal cycles were performed manually. However, in the actual clinical setting, the patients cannot standardize the forces or the direction of their insertion and removal forces which makes the lack of automation of insertion cycles less critical in this context.

On the other hand, a fully guided surgical stent was used to ensure complete implant parallelism and eliminate any misangulation that could have resulted in accelerated wear of retentive inserts and consequently affected the retention [18, 32]. Implants were placed 23 mm apart (11.5 mm away from the midline) as it was found to be the optimum inter-implant distance for retention and consequently the most optimum position to study the wear behavior of retentive inserts.

Thus, the findings of current study can provide a first-step evidence recommendation for the favorable use of PEEK inserts as an attachment system in cases of locator-retained mandibular implant overdentures and should be supplemented by further in-vivo clinical studies.

## Conclusions

Based on the study’s limitations and findings, it can be concluded that PEEK inserts offer superior retention, reduced wear, and increased dimensional stability compared to conventional nylon inserts over various time intervals equivalent to up to 2 years of clinical use following initial prosthesis delivery. Therefore, PEEK is recommended as a preferred prosthetic material for retentive inserts in cases of locator-retained mandibular implant overdentures.

## Data Availability

No datasets were generated or analysed during the current study.
